# Processing and Valorization of Wheat Bran, Germ and Their Fractions: An Evidence-Graded Review of Composition, Technologies and Applications

**DOI:** 10.3390/foods15081455

**Published:** 2026-04-21

**Authors:** Daniela Marisa Ferreira, Ezequiel R. Coscueta, María Emilia Brassesco, Manuela Pintado

**Affiliations:** Universidade Católica Portuguesa, CBQF—Centro de Biotecnologia e Química Fina—Laboratório Associado, Escola Superior de Biotecnologia, Rua Diogo Botelho 1327, 4169-005 Porto, Portugal; dmvferreira@ucp.pt (D.M.F.); mpintado@ucp.pt (M.P.)

**Keywords:** wheat bran, wheat germ, wheat germ oil, enzymatic hydrolysis, fermentation, green processing, bioactive peptides, functional foods, sustainable valorization, circular bioeconomy

## Abstract

Wheat processing generates large volumes of co-products, particularly wheat bran (WB) and wheat germ (WG), which remain underutilized despite their high content of dietary fiber, phenolic compounds, bioactive peptides, and lipophilic antioxidants. Although their composition and processing have been widely investigated, an integrated and application-oriented evaluation of these fractions remains limited. This review provides a structured and critical analysis of WB, raw and defatted WG, and wheat germ oil (WGO), linking composition, processing strategies, and functional performance within a unified framework. Conventional and emerging technologies, including enzymatic hydrolysis, fermentation, thermomechanical treatments, and supercritical CO_2_ extraction, are discussed in terms of selectivity, impact on techno-functional properties, and scalability. An evidence-grading approach is introduced to distinguish bioactivities supported by chemical assays, cell-based models, animal studies, or human data, enabling a more rigorous interpretation of health-related effects. Across applications, these co-products have been incorporated into food systems and related sectors, primarily showing improvements in nutritional composition, oxidative stability, and product performance under experimental conditions. However, translation to an industrial scale remains constrained by techno-economic limitations, regulatory requirements, and stability challenges. This work highlights the need for integrated processing strategies aligned with industrial feasibility to support the development of sustainable cereal biorefineries.

## 1. Introduction

Wheat is among the three most consumed cereals worldwide and was one of the first crops to be cultivated on a large scale, approximately 10,000 years ago [1,2]. At present, it remains a cornerstone of global food systems, providing close to 20% of the world’s caloric and protein intake [3]. According to the Food and Agriculture Organization (FAO), global wheat production reached approximately 801 million tons in 2023, underscoring its significant role in food security and dietary sustainability [3].

Beyond its quantitative relevance, wheat occupies a unique position due to its balanced nutritional profile and its distinctive technological property of forming a viscoelastic dough upon hydration. This functionality underpins the production of a wide range of staple products consumed worldwide [4]. However, the industrial milling process inevitably generates substantial co-products, primarily WB and WG. Although these fractions concentrate a considerable proportion of the grain’s bioactive compounds, they remain underexploited in high-value applications. WB accounts for nearly 150 million tons annually [5], while WG production is estimated at approximately 25 million tons per year, currently directed mainly to animal feeding [6].

Recent reviews have addressed specific dimensions of wheat co-products. For instance, a study examined the functional properties of WB, including antioxidant and cosmetic applications. In contrast, another study (2023) focused on physicochemical modification strategies and highlighted the scarcity of clinical validation [7,8]. Various studies emphasized the underutilized protein fraction of WB [7,8,9]. Regarding WG, it was reviewed protein extraction and peptide bioactivity, while another study discussed polyamines and anti-aging potential [10,11]. Although these contributions are valuable, they predominantly focus on isolated components or specific applications [12]. A holistic, application-oriented integration of composition, processing strategies, techno-functional transformation, and industrial utilization of WB and WG remains limited [13]. From a sustainability perspective, improving the valorization of wheat co-products is both a technological and environmental imperative. WB is still largely directed toward low value uses, such as animal feed or incorporated into foods without targeted functional optimization [14]. WG, despite its rich lipid and protein fractions, is commonly exploited only for oil extraction or marketed in its raw form, where endogenous lipases accelerate oxidative degradation and compromise stability [6,15,16]. These aspects highlight the need for integrated processing approaches that enhance stability, bioactivity, and techno-functional performance.

Despite their compositional richness, the valorization of WB, WG, defatted WG, and WG oil remains constrained by structural and technological bottlenecks. Many bioactive compounds are associated with complex polysaccharide–protein matrices, which may reduce their extractability and bioaccessibility under conventional processing conditions [17]. In addition, the lipid fraction of WG and WG oil contains a high proportion of unsaturated fatty acids, increasing susceptibility to oxidative reactions that can affect stability and sensory attributes during storage. High inclusion levels may further affect texture, processability, and consumer acceptance in food systems [17]. These constraints indicate that the challenge lies not in the absence of valuable compounds, but the difficulty in effectively transforming and stabilizing them for functional applications [17].

**Figure 1:**
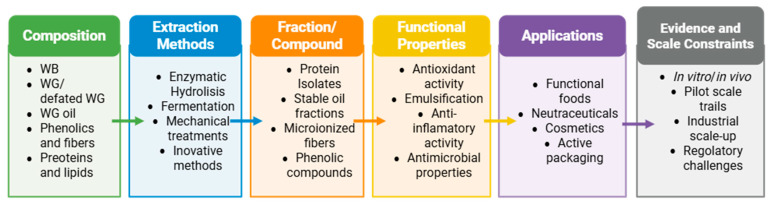
Conceptual framework for the structured evolution of the wheat co-product valorization pathways.

This review provides a comprehensive and critical evaluation of WB, WG, defatted WG, and WG oil through an integrative framework linking composition, processing strategies, structural modifications, and resulting techno-functional and biological properties (Figure 1). In contrast to previous reviews that predominantly addressed isolated components, specific processing techniques, or individual application sectors, the present work adopts a system-oriented perspective, integrating multiple co-product streams and processing routes within a biorefinery context. This approach enables a more holistic understanding of how processing strategies influence the transformation of wheat co-products into functional ingredients. While significant advances have been achieved at the laboratory scale, the transition toward industrial implementation remains limited by techno-economic constraints, process scalability, and regulatory requirements [10,15,18,19,20,21].

Furthermore, this review introduces an evidence-grading framework to contextualize reported bioactivities according to the level of experimental validation, ranging from in vitro assays to human intervention studies. At the same time, it explicitly considers scalability constraints, techno-economic feasibility, and regulatory aspects, which are often underrepresented in the existing literature. By combining mechanistic insight with application-oriented evaluation, this work aims to bridge the gap between laboratory-scale research and industrial implementation, supporting the development of sustainable, resource-efficient, and functionally optimized cereal-based systems.

## 2. Wheat Co-Products: Composition and Potential

**Figure 2:**
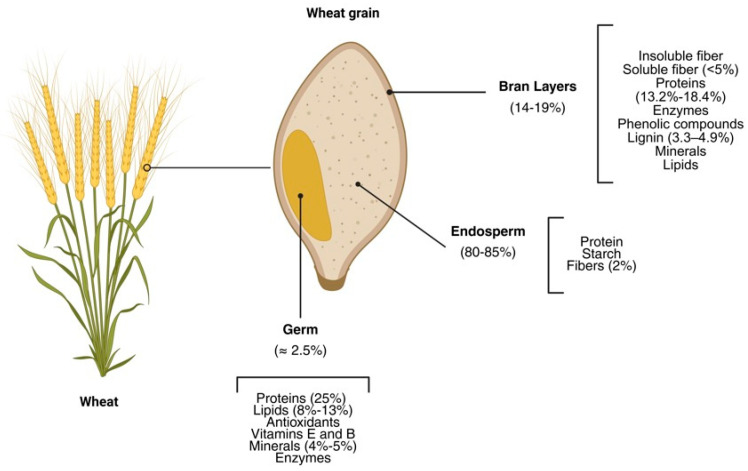
Schematic representation of the approximate compositional ranges of wheat grain fractions (bran, endosperm and germ), based on data reported in [6,25]. Values represent typical intervals reported in the literature and may vary depending on genotype, agronomic conditions, and analytical methodology.

Wheat grain contains a wide array of valuable compounds that are unevenly distributed among its anatomical fractions: the endosperm, the outer layers, and the germ, the latter two constituting the main co-products of the milling process. Industrial wheat milling is designed to separate these anatomical components to obtain refined flour with improved technological stability. This process involves sequential mechanical operations, including cleaning, tempering, grinding, and sieving, which progressively detach the bran layers and the germ from the starchy endosperm [22]. During milling, the endosperm is reduced to fine particles, forming refined flour. In contrast, WB originates primarily from the outer pericarp and aleurone layers removed during the initial breaking and sifting stages. WG is separated at later grinding steps, when the embryo is detached from the endosperm matrix. Although these fractions are excluded to enhance flour shelf life and prevent lipid oxidation, they concentrate a substantial proportion of the grain’s proteins, dietary fibers, lipids, minerals, and bioactive compounds (Figure 2), making them particularly relevant for targeted valorization strategies [22,23].

In industrial practice, wheat co-products such as wheat bran and wheat germ are generated in large volumes as side streams of flour production, primarily due to their negative impact on flour stability and shelf life. While these fractions have traditionally been directed toward low-value applications such as animal feed, their compositional richness has increasingly positioned them as valuable raw materials within emerging cereal biorefinery concepts. In this context, wheat processing is progressively shifting toward integrated valorization strategies, where multiple fractions are simultaneously recovered and transformed into functional ingredients, rather than being treated as residual by-products. This transition reflects growing industrial interest in resource efficiency, waste reduction, and the development of high-value applications from existing agro-industrial streams [24]. Given their distinct structural organization and compositional complexity, WB and WG require individual consideration, as discussed in Section 2.1 and Section 2.2.

### 2.1. Wheat Bran

WB represents approximately 14–19% of the grain weight and is particularly enriched in dietary fiber, essential micronutrients, and bioactive compounds associated with reported physiological effects, including the modulation of gut fermentation and fecal bulk [14,26]. However, the functionality of these components is strongly influenced by their structural organization within a complex and rigid cell wall matrix. Reported compositional ranges are influenced by wheat genotype, agronomic conditions, and milling fraction purity, as well as by the analytical methods employed, particularly for non-starch polysaccharide quantification and phenolic extraction protocols [14,26].

The fiber fraction is predominantly composed of non-starch polysaccharides (NSPs), which are classified by molecular size, structural features, solubility, and fermentability. Arabinoxylans (AX) are the major fraction, accounting for approximately 70% of total NSP, followed by cellulose (≈19%) and β-glucans (≈6%). AX content typically ranges from 5 to 27%, depending on genotype and environmental conditions. Structurally, AX consists of a β-(1→4)-linked xylan backbone substituted with arabinose residues; the arabinose-to-xylose ratio is commonly used as an indicator of branching degree and solubility. β-Glucans occur in lower amounts (<3% dry matter) and contain mixed β-(1→3) and β-(1→4) linkages, contributing to viscosity-related physiological effects [8,27]. Cellulose, primarily located in the outer pericarp, provides mechanical strength, whereas minor pectic polysaccharides contribute to water-binding and gel-forming properties relevant in food matrices [8].

WB is also a significant source of phenolic compounds, most of which are covalently bound to cell wall polysaccharides via ester linkages. Total polyphenol content ranges from 1.4 to 11.1 mg gallic acid equivalents (GAE)/g dry weight, with substantial variability influenced by genetic and environmental factors [28,29]. It should be noted that the total phenolic content is commonly determined using the Folin–Ciocalteu method, which may overestimate values due to interference from non-phenolic reducing compounds [30]. The phenolic profile is dominated by hydroxycinnamic acids, particularly ferulic, sinapic, and p-coumaric acid, alongside minor flavonoids. Ferulic acid, the predominant phenolic constituent, is largely esterified to arabinoxylans, contributing to both structural cross-linking within the cell wall and antioxidant capacity [8]. Lignin (≈8–15%) further reinforces this structural rigidity through ferulate-mediated associations with hemicelluloses. In addition, alkylresorcinols, amphiphilic phenolic lipids, contribute to the antioxidant, antimicrobial, and anti-inflammatory properties attributed to WB consumption [8].

The physiological effects of WB arise from the interplay between its physicochemical properties and microbial fermentation in the colon. Insoluble fractions, such as cellulose and poorly soluble AX, increase fecal bulk and accelerate intestinal transit, whereas soluble fractions modulate digesta viscosity, delay gastric emptying, and attenuate postprandial glycemic and lipidemic responses [31]. In the colon, AX and β-glucans are selectively fermented by the microbiota, leading to the production of short-chain fatty acids (SCFAs) that contribute to improved metabolic regulation, immune modulation, and satiety by stimulating the release of gut-derived hormones [32,33]. Importantly, AX structure, degree of substitution, and molecular weight strongly influence fermentability and bioactivity, while high-molecular-weight β-glucans further enhance viscosity-mediated effects on nutrient absorption [34].

Collectively, the compositional richness of WB supports its classification as a functional ingredient with reported functional relevance in digestive and metabolic models. Nevertheless, the predominance of bound phenolics and the structural rigidity of its fiber matrix limit the immediate bioaccessibility of bioactive compounds, thereby underscoring the importance of targeted processing strategies to enhance functional performance.

### 2.2. Wheat Germ

WG is characterized by a highly concentrated nutritional profile, containing approximately 8–13% lipids, 25% proteins, 17% carbohydrates, 1.5–4.5% dietary fiber, and 4–5% minerals (Figure 1) [17,35]. As the embryonic component of the grain, WG is metabolically active and therefore enriched not only in macronutrients but also in endogenous enzymes and bioactive compounds. Variations in reported compositional values largely reflect differences in germ purity, degree of endosperm contamination, and extraction methodology.

Its lipid fraction is particularly notable due to its content of tocopherols (especially vitamin E) and phytosterols, investigated in oxidative and lipid metabolism models. The vitamin profile is further enriched in B-complex vitamins, including thiamin and riboflavin. In milling-derived germ fractions, carbohydrate content may reach up to 45% of the total constituents; however, this proportion largely reflects residual contamination from endosperm starch and bran-derived polysaccharides rather than intrinsic germ carbohydrates [36,37].

From a functional perspective, WG supplementation has been associated with improvements in metabolic health markers, including reductions in insulin levels, glycated hemoglobin, homeostatic model assessment of insulin resistance, and pro-inflammatory adipokines such as resistin [38]. WG also provides dietary fiber, primarily arabinoxylans and β-glucans, which modulate the gut microbiota and may reduce the risk of obesity and type 2 diabetes [39]. Nevertheless, the bioaccessibility and stability of these compounds depend strongly on processing conditions and matrix interactions. In industrial practice, WG is commonly processed into two main fractions: defatted WG (after oil extraction) and WG oil, due to their distinct compositional profiles and technological behaviors.

#### 2.2.1. Wheat Germ Oil

WG oil can be recovered through mechanical pressing or solvent-based extraction, the latter generally providing higher lipid yields [18]. More sustainable alternatives, such as supercritical CO_2_ (SC-CO_2_) extraction, have emerged as efficient approaches that achieve comparable yields while avoiding toxic solvent residues [40].

However, oil yield and quality are strongly influenced by germ purity, contamination with bran or endosperm, and processing conditions. Despite its high nutritional value, WG oil presents significant technological challenges. Crude oil is typically dark in color and may exhibit intense odor and flavor, largely due to its susceptibility to oxidative degradation. Stabilization and mild refining are therefore essential to preserve quality; nevertheless, conventional refining processes often result in substantial losses of bioactive compounds, particularly tocopherols [41]. This trade-off between oxidative stability and bioactive retention represents a technological limitation due to oxidation-induced losses in tocopherols and sensory deterioration.

From a compositional standpoint, WG oil is characterized by a high proportion of triglycerides rich in unsaturated fatty acids, predominantly linoleic acid, followed by palmitic and oleic acids, with unsaturated lipids accounting for nearly 80% of total fatty acids. Fatty acid profiles may vary depending on genotype, cultivation conditions, storage, and extraction method and are typically determined by gas chromatography following methyl esterification, and minor discrepancies between studies may arise from differences in extraction and derivatization protocols [42]. In addition to neutral lipids, minor polar fractions such as phospholipids are present [18]. WG oil is also an exceptionally rich source of tocopherols and carotenoids, which confer antioxidant capacity, alongside bioactive alcohols including policosanols and phytosterols associated with cholesterol-lowering effects [43,44].

Collectively, these compositional attributes position WG oil characterized by approximately 80% unsaturated fatty acids and high tocopherol levels [43,44], with significant potential in food and nutraceutical applications. However, its high degree of unsaturation simultaneously renders it prone to oxidation, underscoring the importance of optimized extraction, stabilization, and encapsulation strategies, as discussed in subsequent sections.

#### 2.2.2. Defatted Wheat Germ

The extraction of WG oil yields defatted WG as its main co-product, a fraction particularly enriched in proteins, accounting for approximately 35% of its composition [45]. Protein content may vary depending on extraction efficiency and residual lipid removal, as well as on the analytical method used for nitrogen quantification (e.g., Kjeldahl vs. Dumas). These proteins are predominantly albumins and globulins, followed by glutelins and prolamins. They are characterized by a well-balanced amino acid profile with a high proportion of essential amino acids, notably lysine. This composition supports the use of defatted WG as a plant-based protein source for nutritional fortification.

Beyond its protein fraction, defatted WG contains carbohydrates, including soluble sugars, pentosans, dietary fiber, and residual starch, largely due to endosperm contamination during milling [22]. It also retains bioactive compounds, including carotenoids and flavonoids, as well as essential minerals such as potassium, magnesium, calcium, zinc, and manganese [22]. The phenolic fraction exhibits significant antioxidant capacity and has been associated with antimicrobial activity, supporting its potential use in nutraceutical formulations targeting oxidative stress and related disorders [46,47].

From a technological standpoint, defatted WG proteins demonstrate favorable functional properties, including emulsifying and foaming capacity, water-holding ability, and structural stability, making them suitable for incorporation into cereal-based and formulated food systems [36]. Additionally, enzymatically derived WG peptides have been linked to gut-protective and potential anti-aging effects, further expanding the functional scope of this co-product [10]. Collectively, these attributes highlight defatted WG as a high-value protein-rich fraction whose effective valorization depends on targeted processing strategies to enhance bioaccessibility and techno-functional performance.

## 3. Processing Strategies for the Valorization of WB and WG

While WB and WG are intrinsically rich in bioactive compounds and nutrients, their effective utilization depends largely on the application of targeted processing strategies. The structural complexity of cell wall matrices, the limited bioaccessibility of bound phytochemicals, and the susceptibility of lipid fractions to oxidation represent key technological barriers that must be addressed to enable high-value applications.

Processing technologies, therefore, play a dual role: facilitating the release of bioactive components and improving techno-functional performance while preserving nutritional integrity. The selection of an appropriate strategy depends on the intended fraction (WB, raw WG, defatted WG, or WG oil) and the targeted functional outcome.

For clarity, this section is structured according to the main wheat co-products and their respective transformation routes. The compositional characteristics of wheat bran and wheat germ are summarized in Table 1, highlighting their high content of dietary fiber, proteins, and bioactive compounds. The main processing strategies and their effects on target compounds and functional properties are summarized in Table 2 and Table 3 addresses the raw WG and defatted WG. Within each subsection, both conventional and emerging technologies are critically discussed with respect to efficiency, scalability, sustainability, and industrial feasibility.

### 3.1. Wheat Bran Valorization

The processing of WB has been extensively investigated to overcome the structural constraints imposed by its lignocellulosic matrix and to enhance the recovery of dietary fibers, phenolic acids, and protein-derived bioactives. Due to the strong association between phenolics and cell wall polysaccharides, effective valorization strategies must address both matrix disruption and compound stabilization.

Enzymatic hydrolysis remains one of the most selective and controllable approaches. Enzymes such as α-amylase (EC 3.2.1.1) and alkaline proteases have been applied to increase the release of reducing sugars and bound phenolic compounds [25]. At the same time, glycosidases (e.g., Ultraflo XL) enhanced the solubility and antioxidant capacity of ferulic acid-rich fractions [48]. Process intensification through combined technologies, including high hydrostatic pressure (HHP), has further improved phenolic bioaccessibility [50]. However, enzyme performance is highly dependent on substrate accessibility, particle size, and prior structural modification, and enzyme cost may limit large-scale implementation. Moreover, variations in reporting extraction yields and bioaccessibility metrics hinder direct comparison across studies.

Fermentation-based strategies, particularly solid-state fermentation (SSF), offer a complementary bioprocessing route. Fungal strains such as Isaria cicadae and Penicillium oxalicum M1816 significantly enhanced ferulic acid release and soluble fiber content, in some cases surpassing commercial enzymatic treatments [51,52]. Hybrid approaches combining autohydrolysis and SSF reported increases in phenolic liberation up to 62.5% [53]. Nevertheless, fermentation processes are inherently time-dependent and sensitive to microbial variability, requiring strict control to ensure reproducibility and industrial scalability.

Thermomechanical treatments, including steam explosion and extrusion, effectively disrupt bran structure, increasing fiber solubility and facilitating the release of antioxidant compounds [54,55]. While these techniques are generally scalable, they involve considerable energy input and may compromise heat-sensitive bioactives if not carefully optimized. Similarly, micronization improves antioxidant activity and compound solubility by reducing particle size [57], yet ultra-fine milling may pose cost and energy-efficiency challenges at an industrial scale.

Energy-assisted technologies, particularly ultrasound-assisted extraction (UAE), have attracted attention for their enhanced mass transfer and reduced solvent demand. UAE combined with alkaline hydrolysis enabled the high recovery of trans-ferulic acid and total phenolics, with demonstrated antioxidant and antimicrobial potential [59]. Despite these advantages, the long-term stability of ultrasound-treated extracts and the economic feasibility of scale-up remain areas requiring further validation.

Overall, no single processing strategy universally outperforms others. Enzymatic and fermentation-based approaches provide higher selectivity for bioactive release, whereas thermomechanical and physical treatments primarily improve structural and techno-functional properties. Future research should prioritize process standardization, life cycle assessment, and techno-economic evaluation to bridge the gap between laboratory-scale optimization and industrial implementation.

### 3.2. Wheat Germ Valorization

#### 3.2.1. Wheat Germ Oil Valorization

WG oil is one of the most valuable fractions derived from WG, yet its high content of polyunsaturated fatty acids renders it particularly susceptible to oxidation. Consequently, extraction strategies must balance lipid recovery efficiency with preservation of bioactive integrity.

Conventional solvent-based methods, including Soxhlet and chloroform/methanol extraction, ensure the near-complete recovery of fatty acids—predominantly linoleic, palmitic, and oleic acids [65]. These methods remain useful for analytical comparison and baseline assessments of composition, but the use of hazardous solvents and long extraction times limits their industrial desirability.

Cold pressing represents a solvent-free alternative that better preserves heat-sensitive compounds. Oils obtained by cold pressing have demonstrated higher in vitro anti-inflammatory activity than their solvent-extracted counterparts, likely reflecting improved retention of tocopherols [44]. However, lower extraction yields and potential variability in oil recovery remain relevant considerations.

Enzymatic pretreatments have been proposed to enhance lipid release by disrupting protein–lipid interactions within the germ matrix. Protease systems, such as alcalase (EC 3.4.21.62), have increased oil recovery by approximately 66.5% [60]. While enzymatic-assisted extraction improves efficiency under mild conditions, enzyme cost, process optimization, and downstream separation steps must be considered when evaluating industrial feasibility.

Among the emerging technologies, supercritical CO_2_ (SC-CO_2_) extraction offers a solvent-free and highly tunable platform. By adjusting pressure and temperature parameters, selective recovery of lipid fractions can be achieved while preserving tocopherols and carotenoids [44]. Compared to conventional hexane extraction, SC-CO_2_ often yields oils with superior oxidative stability and antioxidant capacity. Nevertheless, the requirement for specialized equipment and high capital investment may limit its use in large-scale or high-value applications.

Microwave- and ultrasound-assisted extractions further enhance mass transfer and cellular disruption, reducing extraction time and solvent demand. Although these technologies may improve antioxidant retention, excessive energy input can compromise thermolabile compounds, requiring careful process control [44].

Overall, no single extraction strategy simultaneously maximizes yield, bioactive preservation, cost-efficiency, and scalability. SC-CO_2_ extraction appears particularly suitable for high-value nutraceutical and cosmetic applications where quality and solvent-free processing are prioritized. Cold pressing remains attractive for clean-label food applications, whereas enzymatic pretreatments provide a cost-adjustable intermediate solution. Future studies should integrate techno-economic assessment and oxidative stability monitoring to enable more informed selection of extraction technologies.

#### 3.2.2. Raw Wheat Germ and Defatted Germ

Recent advances in WG processing have focused on unlocking its protein fraction to obtain bioactive peptides with antioxidant, antihypertensive, immunomodulatory, and cytoprotective properties. Enzymatic hydrolysis remains the most widely applied and controllable strategy. Proteases such as alcalase have generated hydrolysates with high ABTS and DPPH radical scavenging activity, Fe^2+^ chelation capacity, and ACE-inhibitory effects, yielding peptides with well-characterized sequences and multifunctional potential [68,69,87]. The degree of hydrolysis (DH) has been shown to directly influence antioxidant activity, although the relationship between peptide size distribution and bioactivity remains matrix dependent.

Fermentation represents a complementary bioprocessing route. Lactic acid bacteria (LAB), including *Lactobacillus plantarum* and *L. acidophilus*, enhance peptide release through endogenous proteolysis and increase antioxidant capacity [84,85]. Co-fermentation systems incorporating *Saccharomyces cerevisiae* have further enabled the production of bioactive metabolites, such as dimethoxybenzoquinone, thereby significantly improving radical scavenging activity [82]. While fermentation offers eco-friendly processing and probiotic integration, variability in microbial metabolism may affect reproducibility and selectivity compared to purified enzyme systems.

In addition to biochemical strategies, microwave-assisted processing has been applied to WG to enhance macronutrient retention and improve technological performance in enriched food formulations such as pasta [86]. However, its impact on peptide bioactivity requires further systematic evaluation.

Solvent and aqueous extraction approaches have also been explored for phenolic and antioxidant recovery, with solvent polarity significantly influencing extract composition and oxidative stability outcomes [46]. These findings highlight the importance of tailoring extraction systems to targeted functional endpoints.

Defatted WG, generated after oil extraction, provides a protein-enriched matrix particularly suitable for peptide production. Enzymatic hydrolysis using proteases such as alcalase, neutrase (EC 3.4.24.28), papain (EC 3.4.22.2), and trypsin (EC 3.4.21.4) has yielded low-molecular-weight peptides with strong antioxidant and immunomodulatory activity [71]. Targeted hydrolysis has also been investigated to reduce gluten immunogenicity. Microbial peptidases and combined enzymatic systems have significantly decreased coeliac-toxic peptide fractions, demonstrating potential for developing reduced-immunoreactivity ingredients [73]. From a techno-functional perspective, hydrolysis improves solubility, emulsifying capacity, and interfacial stability, with peptide size distribution influencing specific functionalities such as foam stability and emulsification efficiency. Fermentation-based systems further contribute to gliadin degradation, although effects on glutenins remain limited [83].

Overall, enzymatic hydrolysis stands out for its precision and reproducibility in generating multifunctional peptides from both WG and defatted WG. Fermentation provides additional metabolic diversity and potential for reduced immunoreactivity, albeit with lower process control. Integrated approaches combining hydrolysis, fermentation, heat pretreatment, or membrane fractionation have been investigated as strategies to modulate bioactive release and techno-functional properties, with reported process intensities or recovery ranges of 16–30%, depending on the conditions [80]. Future research should prioritize standardization of bioactivity assays, correlation between peptide structure and functionality, and techno-economic validation to support industrial translation.

## 4. Functional and Industrial Applications of Processed Wheat Co-Products

Processed wheat co-products have been incorporated into multiple sectors, including food, cosmetic, nutraceutical, and packaging applications. Their use extends beyond nutritional enrichment, enabling improvements in technological performance, oxidative stability, and controlled release of bioactive compounds. However, successful industrial integration depends not only on compositional enhancement but also on process scalability, regulatory compliance, consumer acceptance, long-term stability, and economic feasibility. Table 4 summarizes the relationship between processing strategies and their resulting functional applications across different sectors.

Although numerous studies report antioxidant, antihypertensive, antimicrobial, and anti-inflammatory effects, most evidence derives from in vitro assays or preclinical models, with limited validation in human intervention studies. As a result, translation into clinically substantiated claims remains constrained [10].

To facilitate interpretation of functional claims discussed in this section, reported findings are contextualized according to a hierarchical evidence framework: Level A, human intervention studies; Level B, animal models; Level C, mechanistic or cellular studies; and Level D, chemical assays (e.g., DPPH, ABTS) or in vitro digestion models. Unless otherwise specified, most bioactivity data for wheat co-products derive from Level C or D evidence.

### 4.1. Food

The incorporation of processed wheat co-products into food systems reflects growing demand for fiber-enriched, protein-enhanced, and functionally optimized formulations.

In cereal-based matrices, these ingredients contribute not only to nutritional upgrading but also to the modulation of digestibility, texture, oxidative stability, and shelf life. However, their technological performance is strongly matrix-dependent and closely linked to prior processing, inclusion level, and particle characteristics.

WB, WG, defatted WG, and WG oil have been integrated into bakery products, pasta, noodles, dairy systems, emulsified sauces, and functional beverages [88,89,94,99]. Reported improvements include enhanced fiber and protein content, modified in vitro starch digestibility (Level D), increased antioxidant capacity measured by radical scavenging assays such as DPPH or ABTS (Level D), and improved structural stability in gluten-based systems assessed through product performance testing. In some cases, metabolic effects such as improved glycemic markers have been explored in animal models (Level B) or small-scale human interventions (Level A); nevertheless, broader clinical validation remains limited. Accordingly, the effectiveness of these incorporations must be evaluated by balancing nutritional enhancement with structural integrity, sensory acceptance, and the strength of supporting biological evidence, particularly in gluten-based systems where matrix interactions play a decisive role.

#### 4.1.1. Wheat Bran Applications

WB incorporation is primarily intended to increase dietary fiber and bioactive compound content in cereal-based systems. However, direct addition of untreated bran frequently compromises dough rheology and sensory quality, leading to reduced loaf volume, weakened gluten structure, impaired gas retention, darker coloration, and coarser crumb texture. These effects arise mainly from bran particles interfering with gluten network formation and competing with gluten for water within the dough matrix [106].

To overcome these limitations, targeted bioprocessing approaches have been developed. Enzymatic treatments, such as cellulase (EC 3.2.1.4) application, modulate fiber structure, and improve water distribution, enabling the production of high-fiber pasta with enhanced cooking performance and sensory acceptance under real processing conditions [88]. Similarly, fermentation of WB with *Auricularia polytricha* improved noodle texture and reduced cooking loss, likely by partially restructuring the gluten–fiber interface and modifying matrix hydration dynamics [89].

More broadly, pre-fermentation of WB using yeast, lactic acid bacteria, or filamentous fungi promotes the partial degradation of complex polysaccharides, increases the release of soluble arabinoxylans and phenolics, and improves dough functionality [106]. These structural modifications translate into enhanced gas cell stability and crumb uniformity in bread, reduced stickiness in noodles and biscuits, and improved flavor development in cakes.

Fermentation has also been associated with reduced in vitro starch digestibility and lower predicted glycemic index values, as determined by enzymatic digestion models (Level D) [90,91,92,106]. While such findings suggest potential modulation of postprandial glycemic response in humans (Level A evidence remains scarce), most evidence derives from laboratory-based digestion assays rather than controlled human intervention studies. Therefore, extrapolation to metabolic outcomes should be interpreted cautiously [92].

Although fermentation improves dough functionality, sensory attributes, and in vitro starch digestibility, most evidence derives from laboratory digestion models (Level D). While these findings suggest potential modulation of glycemic response, controlled human intervention studies remain scarce, and extrapolation to metabolic outcomes should therefore be approached with caution [93,106].

#### 4.1.2. WG Oil

While WG is frequently valorized for its protein fraction, its lipid component presents distinct formulation challenges due to its high degree of unsaturation and susceptibility to oxidation. In food applications, both cold-pressed and emulsified WG oil have been incorporated into cereal and dairy systems. In bread formulations, commercial WG oil improved dough rheology and microbiological stability under storage conditions [100]. In yoghurt systems, emulsion-gels containing WG oil and *Lactobacillus rhamnosus* enhanced pH stability, reduced syneresis, and improved textural attributes, demonstrating compatibility with fermented dairy matrices [94].

To mitigate oxidative instability and expand technological applicability, encapsulation has emerged as a key enabling strategy. Techniques such as spray-drying, freeze-drying, extrusion, ionic gelation, and nanoemulsion formation have been employed to improve oxidative resistance, shelf-life stability, and the retention of tocopherols and phytosterols during storage. The performance of these systems is strongly influenced by wall material selection (e.g., alginate, gum Arabic, pectin, whey or soy protein isolates) and by emulsifier properties that enhance dispersion and limit pro-oxidant interactions within complex food matrices [107].

Recent applications (2019–2025) illustrate the functional versatility of encapsulated WG oil in real food systems. WG oil–starch complexes improved noodle textural stability during storage; nanoemulsion systems enhanced lipid oxidative stability in refrigerated fish fillets; microencapsulation increased vitamin E retention and extended shelf life in fortified cookies compared with liquid oil; and casein-micelle nanoencapsulation improved antioxidant capacity and sensory quality in functional dairy products such as Labneh cheese. In these cases, improvements were primarily demonstrated through oxidative stability assays (Level D), compositional analysis, and product performance testing, rather than through the clinical evaluation of health outcomes (Level A evidence absent) [97,98,107]. Collectively, these findings indicate that encapsulation strategies enable more stable incorporation of WG oil into diverse food matrices, although large-scale processing optimization and long-term storage validation remain necessary for broader industrial adoption.

#### 4.1.3. Raw WG and Defatted WG

Beyond WB, raw WG and defatted WG have been widely investigated as protein-rich fortification ingredients in cereal-based and fermented foods. Due to their high-quality protein profile, essential amino acids (notably lysine), lipid-associated bioactives, and mineral content, their incorporation enhances the nutritional density of bread, pasta, noodles, and baked products [108].

In bread systems, moderate WG inclusion enhances protein and micronutrient content while preserving acceptable loaf structure; however, excessive levels may disrupt dough viscoelasticity and compromise volume. Similarly, in pasta and noodle formulations, WG enrichment increases ash and essential amino acid content without markedly impairing cooking quality when inclusion levels are optimized. Defatted WG has also been successfully applied in macaroni and noodle systems, improving amino acid balance while maintaining protein digestibility under standard processing conditions [102,105,108].

In baked goods, including cookies, cakes, muffins, and biscuits, WG incorporation increases the protein, lipid, mineral, and fiber content. Nevertheless, technological performance is formulation dependent. Particle size plays a decisive role in textural outcomes, with larger WG particles generally associated with reduced cookie hardness. The interaction between dough moisture and particle size significantly influences product color and crumb structure. Importantly, inclusion levels up to approximately 15% have been reported to maintain sensory acceptability when hydration and particle characteristics are properly controlled [103,104].

WG has also been incorporated into fermented dairy and cereal-based beverages, where increases in measured antioxidant capacity (typically assessed by in vitro radical scavenging assays; Level D) and improvements in viscosity and mineral content have been reported [108]. In such systems, fermentation may reduce antinutritional compounds such as phytic acid, contributing to improved mineral bioaccessibility. Applications extend to yoghurt-type beverages, whey-based drinks, traditional fermented foods (e.g., tarhana), roasted WG-based coffee substitutes, and fortified desserts, generally demonstrating dose-dependent technological and compositional effects [101,108].

Although WG and defatted WG exhibit strong potential as multifunctional fortification ingredients, most reported benefits relate to compositional enhancement and techno-functional performance in model or pilot-scale food systems. Systematic evaluation of long-term shelf-life stability, flavor evolution, consumer acceptance, and large-scale processing compatibility remains necessary for broader industrial adoption. Overall, successful integration depends on optimizing inclusion level, particle characteristics, and matrix compatibility to balance nutritional enhancement with structural integrity [104,108].

### 4.2. Other Applications

Beyond food systems, processed wheat co-products have also been explored in cosmetic formulations, sustainable packaging materials, and experimental nutraceutical delivery systems. These applications represent emerging valorization pathways that extend the functional use of cereal-derived bioactives beyond conventional food matrices, although many remain at early stages of technological development.

In cosmetic formulations, ultrasound-assisted alkaline hydrolysates of WB have been incorporated into oil-in-water emulsions exhibiting antioxidant and antimicrobial activity, primarily demonstrated through in vitro chemical and microbiological assays (Level D/C). The high content of trans-ferulic acid and related phenolics contributed not only to oxidative protection but also to improved emulsion stability, highlighting the dual technological and bioactive role of bran-derived extracts in dermocosmetic systems [59].

In sustainable packaging, WG-derived bioactive peptides have been embedded into polylactic acid/ethyl cellulose films, enhancing antioxidant performance and inhibiting the growth of Escherichia coli and Staphylococcus aureus under controlled laboratory conditions [70]. Such approaches demonstrate the feasibility of integrating cereal-derived bioactives into biodegradable polymer matrices, potentially contributing to active packaging solutions designed to improve product stability and extend shelf life.

WG-derived peptides have also been investigated in controlled-release systems. Microencapsulation using starch sodium octenylsuccinate and sodium alginate protected albumin-derived peptides from simulated gastric degradation and enabled sustained intestinal release in in vitro digestion models (84.41% after 2 h) (Level D) [100]. These findings underscore the relevance of structural protection strategies for enhancing peptide stability during gastrointestinal transit; however, confirmation of bioavailability and physiological efficacy in vivo remains limited.

Collectively, these developments indicate that wheat-derived bioactives may contribute to high-value non-food and adjacent applications, particularly in packaging and dermocosmetics. However, industrial adoption will depend on regulatory compliance, toxicological validation, process scalability, and long-term stability assessment. Bridging the gap between laboratory innovation and commercially viable implementation remains a key challenge for the broader valorization of wheat co-products [43].

## 5. Conclusions and Future Perspectives

The valorization of WB, raw and defatted WG, and WG oil represents a strategic opportunity to transform wheat processing from a linear production system into a circular and resource-efficient model. These co-products, traditionally undervalued or directed to low value uses, are in fact concentrated reservoirs of fibers, bioactive peptides, phenolic compounds, and lipophilic antioxidants with demonstrated functional and technological relevance.

Processing technologies have proven essential to unlock this potential. Rather than a single optimal method, the evidence indicates that valorization efficiency depends on aligning processing strategy with the target compound and final application. Enzyme-assisted hydrolysis enables the controlled cleavage of protein and polysaccharide structures, facilitating the release of peptides and phenolic compounds under defined reaction conditions. Fermentation promotes microbial biotransformation and the partial degradation of structural matrices, contributing to modified compositional and techno-functional properties. Thermomechanical and physical treatments alter particle size distribution and matrix organization, influencing hydration, extractability, and functional behavior. Green extraction technologies, such as supercritical CO_2_, allow for the selective recovery of lipid fractions while minimizing solvent residues and thermal degradation. The combination of complementary techniques has been reported to increase the extraction yield and modify functional performance, depending on the process parameters and target compounds. The integration of complementary processes is therefore a key direction for maximizing both yield and functionality within a biorefinery framework [109].

Despite these advances, translation beyond laboratory and pilot scales remains limited. Most studies focus on compositional improvements or in vitro bioactivity, while techno-economic performance, scalability, and regulatory integration are less frequently addressed. In particular, comparative evaluations of processing strategies in terms of cost, energy demand, and operational complexity remain scarce, limiting the identification of optimal routes for large-scale implementation. In this context, the incorporation of techno-economic analysis (TEA) and life cycle assessment (LCA) into future studies will be essential to support decision-making and process optimization [110]. Most reported benefits relate to compositional enrichment, in vitro bioactivity, or improvements in product performance, frequently supported by Level C–D evidence, while robust human intervention studies remain scarce. Furthermore, industrial implementation requires consideration of techno-economic feasibility, large-scale processing compatibility, and long-term storage stability, aspects that are rarely incorporated into early-stage experimental designs. Regulatory integration also represents a critical but underexplored dimension, as commercialization of novel fractions or concentrated extracts may require alignment with Novel Food authorization pathways, substantiation of health claims under European Food Safety Authority frameworks, compliance with allergen and gluten labeling regulations, control of process contaminants, and monitoring of solvent residues where applicable.

Future research should therefore prioritize methodological harmonization and translational relevance. Standardized reporting of phenolic release—clearly distinguishing free and bound fractions, specifying analytical units, and documenting extraction recovery—would greatly improve cross-study comparability. In the case of WGO, the systematic evaluation of oxidative stability using harmonized metrics such as peroxide value, p-anisidine value, total oxidation indices, and tocopherol retention under realistic storage conditions is essential to assess functional durability. For WB and WG fractions intended for bakery and pasta applications, a clearer definition of sensory acceptability thresholds in relation to inclusion level, particle size, and processing history would facilitate practical formulation guidelines. In parallel, comparative life cycle assessment and techno-economic analysis of major processing routes, including solid-state fermentation, enzyme-assisted hydrolysis, and thermomechanical treatments such as steam explosion, are needed to determine environmental and economic viability at the industrial scale. Finally, stronger integration between bioaccessibility models and matrix-specific evaluation, distinguishing chemical antioxidant capacity from physiologically validated outcomes, will be necessary to avoid the overinterpretation of early-stage evidence. In addition, several constraints must be considered for successful industrial integration.

**Figure 3:**
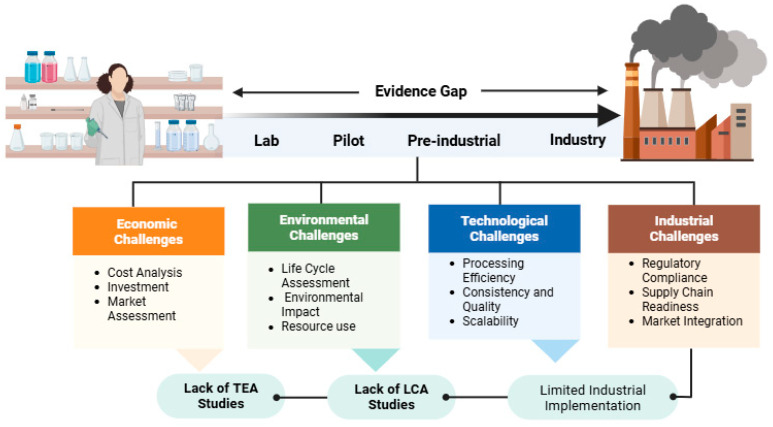
Overview of the techno-economic and scale-up challenges in the valorization of wheat co-products. The transition from laboratory to industrial implementation is hindered by interconnected economic, environmental, technological, and industrial constraints, highlighting the need for integrated techno-economic assessment (TEA) and life cycle assessment (LCA) approaches, based on data reported in [110].

Regulatory requirements, including Novel Food approval, substantiation of health claims under European Food Safety Authority (EFSA) guidelines, and compliance with contaminant and labeling standards, represent significant barriers to commercialization [111]. Product stability during storage, particularly for lipid-rich fractions prone to oxidation, and the preservation of bioactive compounds under processing and distribution conditions remain critical challenges. Sensory acceptance, including effects on texture, flavor, and appearance, must also be carefully balanced with nutritional enhancement to ensure consumer acceptance. In particular, harmonized reporting of bioactive compound release, comprehensive assessment of oxidative stability, and evaluation of sensory thresholds in food systems will be necessary to support industrial translation. These challenges highlight a clear gap between laboratory-scale findings and industrial implementation. As illustrated in Figure 3, the valorization of wheat co-products is constrained by interconnected techno-economic, environmental, technological, and industrial factors.

Overall, wheat co-products should no longer be regarded as secondary outputs of milling but as multifunctional raw materials with the potential to contribute meaningfully to sustainable food systems and high-value functional ingredients. Their effective integration into the bioeconomy will depend not only on technological refinement but also on standardized evaluation frameworks, regulatory alignment, economic validation, and coordinated collaboration between academia and industry. Their effective integration into industrial value chains will depend on the alignment of technological innovation with economic viability, regulatory compliance, and consumer-driven product development.

## 6. Scope and Literature Approach

A structured literature search was conducted to identify relevant publications addressing the composition, processing strategies, and applications of wheat co-products. Four major scientific databases were consulted: PubMed, Scopus, ScienceDirect, and Google Scholar. The search focused on peer-reviewed articles published in English, with priority given to studies published between January 2020 and December 2025 in order to capture recent methodological and technological advances in wheat co-product valorization.

The search strategy combined terms related to wheat fractions with keywords associated with composition, processing, and functional applications. Representative search strings included combinations such as (“wheat bran” OR “wheat germ” OR “wheat germ oil” OR “wheat co-products”) AND (“composition” OR “processing” OR “extraction” OR “valorization” OR “functional properties” OR “bioactivity” OR “applications”) in Scopus, and (“wheat bran” OR “wheat germ” OR “wheat germ oil”) AND (“bioactive compounds” OR “processing technologies” OR “functional properties” OR “food application”) in PubMed. Variations and related terms were iteratively applied to refine results and ensure comprehensive coverage of compositional, mechanistic, and application-oriented studies.

The initial search yielded approximately 350–450 records after the removal of clear duplicates. Titles and abstracts were screened for relevance to wheat bran, raw and defatted wheat germ, and wheat germ oil, with emphasis on studies reporting empirical compositional data, processing mechanisms, techno-functional performance, or bioactivity assessment. Around 170–210 articles were selected for full-text evaluation, of which approximately 115 were ultimately included based on methodological clarity, relevance to the integrative framework adopted in this review, and contribution to understanding composition–processing–application relationships.

Although emphasis was placed on literature published from 2020 onward, earlier foundational studies were included where necessary to provide mechanistic context, define compositional baselines, or describe processing principles that remain relevant to current technological developments.

This review does not follow a formal systematic review protocol, such as PRISMA. Instead, it adopts a structured narrative approach aimed at providing a comprehensive and critical synthesis of the literature while maintaining transparency regarding search strategy and selection rationale.

## Figures and Tables

**Table 1:** Methods of extraction of valuable products from wheat bran.

Method Type	Method/Conditions	Target Compounds	Aim	Main Achievements	Ref.
Enzymatic Hydrolysis	α-Amylase + alkaline proteases (50 °C, pH 10.0, 6 h)	Reducing sugars, xylooligosaccharides, phenolics	To recover sugars and phenolic acids	Hydrolysates rich in phenolics; antioxidant	[25]
	Glycosidases (Ultraflo XL, 47 °C, pH 4.4, 20.8 h)	Phenolic acids	To increase the solubility of phenolic acids	Higher solubility; improved antioxidant and anti-inflammatory properties	[48]
	Viscozyme Cassava C (a multi-enzyme complex containing carbohydrases)	Insoluble fibers	To improve the ratio of insoluble to soluble dietary fibers	Enhanced a lowered ratio of insoluble to soluble dietary fibers When the hydrolysates were introduced in cookies, there was an overall acceptance	[49]
	Enzymatic hydrolysis + High Hydrostatic Pressure (HPP) (60–70 °C)	Phenolic compounds	To enhance bioaccessibility	Increased antioxidant bioaccessibility	[50]
Alkaline Process	NaOH 1 M, pH 9.5, ratio 1:10 (durum WB suspension)	Protein	To extract protein from durum wheat bran	Protein concentrates (61%); durum wheat bran protein concentrate enriched in essential amino acids and phytosterols	[26]
Fermentation	Solid-state (*Isaria cicadae*, *Cordyceps militaris*, *Inonotus obliquus*)	Dietary fiber, flavonoids, phenols	To increase fiber solubility and antioxidant content	Higher soluble fiber, enriched flavonoids & phenolics, improved antioxidant capacity	[51]
Fermentation	Solid-state (*Penicillium oxalicum* M1816)	Ferulic acid	To release ferulic acid	Higher ferulic acid yield compared to commercial enzymes	[52]
	Autohydrolysis pretreatment + solid-state (*Aspergillus niger* and *Eupenicillium parvum*)	Sugars, ferulic acid	To boost fermentable sugars and phenolic release	Ferulic acid recovery up to 62.5%	[53]
Thermochemical	Steam explosion	Dietary fiber, phenolic compounds	To increase soluble fiber & antioxidant activity	Soluble fiber up to 40.32%; improved glucose adsorption and enzyme inhibition	[54]
	Steam explosion + extrusion	Arabinoxylans, phenolic compounds	To improve fiber solubility and release phenolics	Higher arabinoxylan solubility, enhanced in phenolic acids and flavonoid release; antioxidant	[55]
	Alkaline/oxidative vs. hydrothermal processing (160 °C, 6.5 bar)	Arabinoxylans	To recover arabinoxylans in a green process	The yield was superior with the hydrothermal method; lower molecular weight with the alkaline/oxidative extraction compared to the hydrothermal extraction	[56]
Physical (size reduction)	Micronization (particle size 46 → 26 µm)	Proteins, phenolics, fibers	To improve the accessibility of the compounds	Increased solubility; enhanced antioxidant and functional properties	[57]
Energy-Assisted	Ultrasound-assisted	Insoluble fibers	Extract insoluble fibers from wheat bran varieties	Reduction in protein and fats, and highly pure insoluble dietary fibers; Improved cation exchange capacity; improved emulsifying capacity	[58]
	Ultrasound-assisted + alkaline hydrolysis	Phenolic acids	To maximize phenolic release	High yield of trans-ferulic acid and total phenolics; strong antioxidant and antimicrobial activities	[59]

**Table 2:** Methods of extraction of oil from wheat germ.

Method Type	Method/Conditions	Target Compounds	Aim	Main Achievements	Ref.
Enzymatic Hydrolysis	Alcalase 2.4 L FG, ratio liquid/solid 16.5, enzyme conc. 1.1%, 19.25 h (response surface methodology optimized)	Oil fraction	Optimize enzymatic hydrolysis to improve oil yield	Optimal conditions gave 66.5% oil yield	[60]
	Viscozyme L (a multi-enzyme complex containing carbohydrases), Viscoferm; compared to Soxhlet and alkaline hydrolysis (10% KOH)	Fatty acids	Release fatty acids from wheat germ	Highest content of linolenic acid with Viscoferm and Viscozyme L, ensuring 17.0% and 13.6% higher yield than after A-AH, respectively.	[61]
Chloroform/Methanol Process	Follow the method by Folch, J. et al. (1956) [62]	Wheat germ oil	Compare oil processing methods	Fatty acid composition similar across methods; cold pressing gave highest anti-inflammatory activity	[44]
Cold Pressing	Automatic oil presser, 304 SS, 1500 W			Higher anti-inflammatory activity (93.4%, IC_50_ = 195.7 µg/mL) and cyclooxygenase 1 (80.5%, IC_50_ = 58.6 µg/mL) than Soxhlet or chloroform/methanol	
Soxhlet Extraction	Soxhlet, petroleum ether, 6 h			Similar fatty acid profile among methods	
Supercritical CO_2_	4.5 kg WG, 80 °C, 68 MPa, 16 kg CO_2_	Oil fraction	Compare conventional vs. green extraction methods	Conventional methods reduced tocopherol content; Supercritical CO_2_ extraction preserved compounds	[63]
	Using response surface methodology; optimum conditions—35 MPa, 50 °C, 22.5–25 L/h solvent flow, 1 h	Oil fraction	Optimize pressure, temperature, flow rate, time to maximize oil extraction	Quality and yield similar to hexane-extracted oil, but the supercritical CO_2_ uses less solvent	[64]
Soxhlet Extraction	13 g wheat germ, n-hexane + methylene chloride (120 mL), 15 cycles	Fatty acids, antioxidants	Compare methods for yield and fatty acid composition	Supercritical fluid extraction achieved the highest yield, tocopherol content, and antioxidant activity	[65]
Microwave-Assisted Extraction	1:10 *w*/*v* wheat germ:solvent, 360 W, 30 min, solvents n-hexane or methylene chloride				
Ultrasound-Assisted Extraction	Sonication bath, 1:10 *w*/*v*, 40 kHz, then solvent extraction with n-hexane or methylene chloride				
Supercritical Fluid Extraction	70 g wheat germ; pressures 250–350 bar, temperatures 40–60 °C, CO_2_ flow 0.2–0.4 kg/h				
Aqueous Process	Sodium borate pH 8.0, Tris-HCl pH 8.0, citrate-phosphate pH 5.0	Oil fraction	Evaluate buffer/pH effects on processing yield	Higher yield at alkaline pH (Tris-HCl, sodium borate)	[66]

**Table 3:** Methods of extraction of protein and bioactive peptides from raw wheat germ and defatted wheat germ.

Method Type	Method/Conditions	Target Compounds	Aim	Main Achievements	Ref.
Enzymatic Hydrolysis	Alcalase hydrolysis at 52.3 °C, pH 8.0, 223 min; enzyme/substrate ratio 1.46%	Defatted wheat germ proteins	Optimize hydrolysis; identify active fractions	High antioxidant activity (ABTS, DPPH, Fe^2+^ chelation); bioactive peptides identified (GNPIPREPGQVPAY, TVGGAPAGRIVME)	[67]
	Alcalase hydrolysis at 50 °C, pH 8.0, 6 h; enzyme/substrate ratio 0.4 AU/g protein	Defatted wheat germ proteins	Evaluate antioxidant potential	Antioxidant activity (DPPH, superoxide, hydroxyl radicals); inhibition of lipid oxidation; molecular weight distribution ~1500 Da	[68]
	Pepsin (EC 3.4.23.1) (37.4 °C, pH 3, 270 min), Alcalase (52.3 °C, pH 8, 233 min), Proteinase K (37 °C, pH 8, 285 min)	Wheat germ proteins	Assess antioxidant, ACE-inhibitory and cytotoxic activity	Peptides with antioxidant (KELPPSDAW), ACE-inhibitory (SGGSYADELVSTAK), cytotoxic (SSDEEVREEKELDLSSNE) activity identified	[69]
	Protein hydrolysates prepared with Alcalase (10% *w*/*v* protein; 52 °C; pH 8; 4 h; E/S = 1.5%)	Defatted wheat germ bioactive peptides	Produce a film coating with WG peptide hydrolysates and test antimicrobial/antioxidant activity	The film coated with hydrolyzed wheat germ peptides had the highest antioxidant activity and reduced the growth of *Escherichia coli* and *Staphylococcus aureus*	[70]
	Multi-enzyme hydrolysis (pepsin, trypsin, papain, neutrase, alcalase; optimal conditions)	Globulin proteins	Improve immunomodulatory properties	Highest hydrolysis degree with alcalase/neutrase; strong immunomodulatory activity; hydrophobic peptides 300–1450 Da	[71]
	Protease from *Withania coagulans* (45 °C; pH 5; 6 h)	Wheat gluten peptides	Evaluate antioxidant and ACE-inhibitory activities	Two fractions showed strongest bioactivities: F1 (<3 kDa) had the highest antioxidant and F2 (3–30 kDa) had the highest ACE-inhibitory	[72]
	Hydrolysis with peptidases from *L. acidophilus* 5e2 and *A. niger* (pH 4–6, 30–37 °C, 3 h, 100 U/mg enzyme)	Gliadins, coeliac-toxic peptides	Reduce immunoreactivity of gluten peptides	Immunoreactivity reduced to 0.08 µg/mg; key peptides decreased 126- and 31-fold, improving suitability for food biotechnology	[73]
Enzymatic Hydrolysis	Hydrolysis with alcalase (E/S 0.1 AU/g, 50 °C, pH 8, 60 min) in 10% gluten *w*/*v*	Wheat gluten proteins	Investigate immunomodulatory and antioxidant properties (peripheral blood mononuclear cells, ex vivo)	Reduced pro-inflammatory cytokines (Th1/Th17), enhanced antioxidant capacity, increased glutathione	[74]
	Hydrolysis with alcalase, Flavourzyme (a commercial protease mixture), savinase (EC 3.4.21.62), subtilisin (50–60 °C, pH 7–9, E/S 1%, 270 min)	Glutenin fractions	Evaluate effect of enzyme type on degree of hydrolysis and bioactivity	Savinase and subsitilin hydrolysates had the lowest molecular weight peptide fractions as the degree of hydrolysis increased; hydrolysates showed antioxidant, anti-diabetic, anticancer activities; higher degree of hydrolysis → stronger effects	[75]
	Wheat proteins modified at 30 °C, 3 h, with prolyl endopeptidase (EC 3.4.21.26), transglutaminase (EC 2.3.2.13) or peptidases (*L. acidophilus*, *L. sanfranciscensis*); single and two-step treatments	Glutenins, ω-gliadins	Assess immunoreactivity	Two-step treatments reduced immunoreactivity most; transglutaminase/peptidase (*L. sanfranciscensis*) and peptidase (*L. sanfranciscensis*)/transglutaminase were the best combinations	[76]
	Acid protease hydrolysis optimized by response surface methodology (48.9 °C, pH 3.0, E/S 1.6%, 2.5 h)	Wheat gluten	Enhance emulsifying activity and functional properties	Peptides < 10 kDa; conformational changes (FTIR) contributed to better emulsifying properties	[77]
	Gluten hydrolysis optimized by Box–Behnken (1–9% *w*/*v*, 40–60 °C, pH 7–9, E/S 0.25–0.75 AU/g, 2 h)	Wheat gluten proteins	Assess effect of process parameters on degree of hydrolysis and antioxidant properties	Higher degree of hydrolysis→ higher DPPH activity; ABTS activity independent of degree of hydrolysis, suggesting larger bioactive peptides	[78]
	Hydrolysis with papain (1500 U/g) at 50 °C, pH 6.5, 8 h, 8% gluten *w*/*v*), 10 min pre-incubation	Wheat gluten proteins	Evaluate changes in nitrogen forms, degree of hydrolysis and peptide length	Decreased peptide chain length, increased soluble nitrogen; peptides > 15 kDa decreased, <5 kDa increased; protein (gliadin) and soluble glutenin were prone to enzymatic hydrolysis	[79]
Assisted Enzymatic Hydrolysis	Enzymatic hydrolysis heat-assisted: Heat pretreatment (75 °C, 30 min; equilibration 60 °C, 20 min) + Alcalase (E/S 0.5 AU/g, pH 8)	Wheat gluten	Enhance solubility, antioxidant and functional properties	Hydrolysates (degree of hydrolysis 16–30%) showed excellent solubility (pH 2–12), improved emulsification and antioxidant activity (DPPH, ABTS)	[80]
	Enzymatic hydrolysis + ultrafiltration: Hydrolysis of wheat gluten (8% *w*/*v*) at 48 °C, pH 6.8, E/S 2000 U/g + fractionation (ultrafiltration)	Wheat gluten proteins	Improve solubility, emulsifying and foaming properties	Increased solubility across pH 2–10; 50 kDa fraction improved emulsification; 100 kDa fraction yielded most stable foam (65.8%)	[81]
Fermentation	*Saccharomyces cerevisiae* + *L. plantarum*; optimized by response surface methodology	Wheat germ phenolics, peptides, dimethoxy benzoquinone (DMBQ)	Explore and determine the optimum fermentation conditions of WG	Increased phenolics (3.33 mg GAE/g), release of DMBQ (0.56 mg DMBQ/g), DPPH scavenging (86.49%)	[82]
	Sourdough fermentation with lactic acid bacteria (*L. alimentarius*, *L. brevis*, *L. sanfranciscensis*, *L. hilgardii*) (200 g flour, 37 °C, 4–8 h, pH 4.3–4.6)	Albumins, globulins, gliadins	Evaluate proteolytic activity of lactic acid bacteria and effect on gliadin toxicity	Lactic acid bacteria hydrolyzed albumins, globulins, gliadins (not glutenins); degraded toxic α-gliadin (31–43); reduced immunotoxicity	[83]
Fermentation	*Lactobacillus plantarum* (10^8^ CFU/mL, 37 °C, 18 h)	Wheat germ protein	Extract peptides by lactic acid bacteria fermentation	Enhanced proteolytic activity; hydrophobic amino acid peptides; strong antioxidant and radical scavenging activity; development of gluten-sensitive-friendly ingredient	[84]
	*L. acidophilus* + *L. plantarum* (0.5–20% wheat germ, 37 °C, 24 h); electrospraying	Peptides + probiotics	Develop non-dairy probiotic powders	Stable powders with antioxidant activity and probiotic viability	[85]
Solvent/Aqueous Process	Defatted wheat germ treated with water, 30–100% ethanol	Bioactive peptides	Assess the antioxidant potential of the peptides obtained with different solvents	100% ethanol → strongest ABTS; 50–70% ethanol → antioxidant activity in linoleic acid system; phenolics 13.98–16.75 mg GAE/g	[46]
Microwave	700 W, 12 min, 110 °C (intermittent mixing)	Wheat germ proteins, macronutrients	Produce an enriched macaroni	Wheat germ-enriched macaroni with higher protein, fat, carbohydrate content; acceptable storage/cooking quality; slight acidity increase in cooking water	[86]

**Table 4:** Methods of application of valorized wheat co-products.

Co-Product	Processed Form	Obtained Product	Sector	Key Added Value	Ref.
Wheat Bran	Ultrasound-assisted alkaline hydrolysates	Oil-in-water cosmetic emulsion	Cosmetics	High content of *trans*-ferulic acid (406.14 ± 0.65 μg FA/mg extract), total phenolics (610.58 ± 57.60 mg GAE/g dried extract); antioxidant/antimicrobial extract supports stable oil in water cream	[59]
	Cellulase-treated wheat bran	High-fiber pasta	Food	Enhanced cooking properties and sensorial characteristics of pasta	[88]
	Fermented wheat bran (with *Auricularia polytricha*)	Enriched noodles	Food	↑ Noodle hardness; ↓ cooking loss vs. unfermented wheat bran; more favorable gluten network	[89]
	Fermented wheat germ with lactic bacteria	Bread	Food	Increased antioxidant activity and soluble dietary fiber content. Improved composition and functional properties	[90]
	Fermented wheat bran (liquor koji and yeast)	Noodles	Food	Reduced the cooking time and increased the storage stability	[91]
	Fermented wheat bran with *Leuconostoc citreum* TR116	Biscuit	Food	Improved dough quality and sensory properties	[92]
	Fermented wheat bran (solid-state fermentation by *Rhizopus oryzae*)	Cake	Food	Enhanced nutritional and sensory properties. Improved digestibility	[93]
WG Oil	Commercial wheatgerm oil by De Wit Specialty oils BV (Netherlands)	Bread formulations	Food	Impacts dough properties and microbiological quality	[94]
	Supercritical CO_2_ extracted wheat germ oil	Phyto-nanoemulsions	Cosmetic/nutraceutical	Cell-based antioxidant capacity; wound healing	[95]
	Emulsified wheat germ oil	Yoghurt fortified with wheat germ oil emulsion-gel + *L. rhamnosus*	Food	Improved pH and acidity stability during storage; reduced syneresis; all texture profile parameters enhanced	[96]
Wheat Germ Oil	Wheat germ oil nanoemulsions	Cooked fish	Food	The cooked fish loaded with wheat germ oil nanoemulsions had a higher shelf life, and some nutritional parameters were improved, such as the concentration of polyunsaturated fatty acids	[97]
	Wheat germ oil nano-encapsulated (ultrasound)	Labneh cheese	Food	Improves the oxidative stability, the composition, and functional quality of the cheese	[98]
Wheat Germ	Microwaved wheat germ	Macaroni formulation	Food	Acceptable storage; improved nutritional profile	[86]
	Wheat germ and wheat germ protein isolate	High-protein pasta with an improved amino acid composition	Food	The addition of 25% wheat germ and 12% wheat germ protein isolate increased the protein content in the pasta by 35% and 53%; and both improved the amino acid composition	[35]
	Wheat germ protein isolate	Low-cholesterol mayonnaise	Food	Able to produce a low-cholesterol mayonnaise with similar properties to regular ones using a combination of wheat germ protein isolate, xanthan, and egg yolk	[99]
	Wheat germ bioactive peptides	Active polylactic acid/ethyl cellulose film	Packaging	Films coated with wheat germ peptides had the highest antioxidant activity; reduce the growth of Gram-negative bacteria (*Escherichia coli*) and Gram-positive bacteria (*Staphylococcus aureus*) when compared with films coated with chitosan	[70]
Wheat Germ	Microencapsulation (starch sodium octenylsuccinate: sodium alginate = 3:1; freeze-drying) of wheat germ albumin polypeptides	Microencapsulated wheat peptides	Nutraceutical	Embedding rate 65.33%; 2 h release: 26.54% (simulated gastric) and 84.41% (simulated intestinal); FTIR/TGA show protection; mean size ~100 µm	[100]
	Raw wheat germ	Coffee substitute	Beverage	A high quality coffee substitute can be prepared from WG and suitable edible plants	[101]
	Raw wheat germ	Bread	Food	Improved the quality of the Chinese steamed bread. Enhanced the sensory acceptability and physicochemical properties	[102]
	Coarse and fine wheat germ + emulsifier	Cake	Food	Coarse wheat germ gave better physical properties than fine wheat germ. Coarse wheat germ did not change nutritional composition. Wheat germ with sodium stearoyl-2 lactylate were the best combination to improve physical and sensory properties	[103]
	Defatted wheat germ	Cookies	Food	It is possible to add wheat germ up to 15% without altering the sensory characteristics and improving dough quality and nutritional composition	[104]
	Raw wheat germ + β-glucan	Turkish noodles	Food	Wheat germ + β-glucan improved the nutritional value of the noodles, but in higher concentrations negatively affected the sensory quality	[105]

## Data Availability

No new data were created or analyzed in this study.

## Abbreviations

The following abbreviations are used in this manuscript:

ABTS	2,2′-Azino-bis(3-ethylbenzothiazoline-6-sulfonic acid)
ACE	Angiotensin-Converting Enzyme
AX	Arabinoxylans
DH	Degree of Hydrolysis
DMBQ	Dimethoxy Benzoquinone
DPPH	2,2-Diphenyl-1-picrylhydrazyl
FTIR	Fourier Transform Infrared Spectroscopy
GAE	Gallic Acid Equivalent
HHP	High Hydrostatic Pressure
LAB	Lactic Acid Bacteria
NSPs	Non-Starch Polysaccharides
PUFAs	Polyunsaturated Fatty Acids
SC-CO_2_	Supercritical fluid CO_2_
SCFAs	Short-Chain Fatty Acids
SSF	Solid-State Fermentation
UAE	Ultrasound-Assisted Extraction
WB	Wheat Bran
WG	Wheat Germ
